# Transcriptomic and Metabolomic Basis of Short- and Long-Term Post-Harvest UV-C Application in Regulating Grape Berry Quality Development

**DOI:** 10.3390/foods10030625

**Published:** 2021-03-16

**Authors:** Kekun Zhang, Wanping Li, Yanlun Ju, Xianhang Wang, Xiangyu Sun, Yulin Fang, Keqin Chen

**Affiliations:** College of Enology, Northwest A&F University, Yangling 712100, China; zhangkekun1990@nwafu.edu.cn (K.Z.); liwanping@nwafu.edu.cn (W.L.); juyanlun2016@nwafu.edu.cn (Y.J.); Wangxianhang@nwafu.edu.cn (X.W.); sunxiangyu@nwafu.edu.cn (X.S.); fangyulin@nwafu.edu.cn (Y.F.)

**Keywords:** post-harvest, UV-C, Grape, transcriptome, metabolome

## Abstract

In this study, ultraviolet-C (UV-C) was utilized to improve the quality of post-harvest grape berries, and the transcriptomic and metabolomic basis of this improvement was elucidated. Berries of the red grape variety ‘Zicui’ and the white variety ‘Xiangfei’ were chosen to evaluate the effect of short- and long-term UV-C irradiation. Post-harvest UV-C application promoted malondialdehyde (MDA) and proline accumulation, and reduced the soluble solid content in berries. Both the variety and duration of irradiation could modulate the transcriptomic and metabolomic responses of berries to UV-C. Compared with the control, the differentially expressed genes (DEGs) identified under UV-C treatment were enriched in pathways related to metabolite accumulation, hormone biosynthesis and signal transduction, and reactive oxygen species (ROS) homeostasis. Flavonoid biosynthesis and biosynthesis of other secondary metabolites were the shared pathways enriched with differential metabolites. After long-term UV-C irradiation, cis-resveratrol accumulated in the berries of the two varieties, while the differential chalcone, dihydroflavone, flavonoid, flavanol, and tannin components primarily accumulated in ‘Xiangfei’, and some flavonols and anthocyanins primarily accumulated in ‘Zicui’. Based on an exhaustive survey, we made a summary for the effect of UV-C in regulating the quality development of post-harvest grape berries. The results of this study may help to elucidate the mechanism by which UV-C functions and support its efficient application.

## 1. Introduction

Grapes are rich in a variety of resources that can be utilized not only in the fresh fruit supply but also in the production of raisins or wine, continuously meeting the various needs of consumers worldwide. Grape berries possess high antioxidant activity due to their high levels of polyphenols and flavonoids, which is of strong significance for maintaining human health and preventing the occurrence of cardiovascular diseases [1,2].

To improve the health benefits of grape berries, producers generally regulate berry quality development and attempt to increase the content of nutrient components at the on-vine stage and post-harvest stage. In the on-vine stage, various cultivation techniques were evaluated for their effect on quality development. Light quality can modulate the biosynthesis of flavonoids. Ultraviolet light filtering from the flowering stage was observed to significantly inhibit the biosynthesis of flavonols, while visible light filtering was determined to specifically affect anthocyanin accumulation [3]. Hormone signals are closely related to the ripening process of fruit therefore, the spraying of exogenous hormones and their analogues, such as ABA, strigolactone, and MeJA, was applied to regulate the formation of quality traits [4,5]. During the post-harvest stage, many techniques have also been evaluated as a means of promoting the accumulation of secondary metabolites. Post-harvest treatment with ultraviolet-B (UV-B) and UV-C radiation increased the content of phenolic acid components in grape berries by activating the expression of key genes in the phenylpropanoid biosynthesis pathways, and the effect of UV-C was more notable [6]. Meanwhile, suitable post-harvest light conditions (white with ultraviolet light) combined with the optimal temperature (15–20 °C) was observed to upregulate the expression of genes related to anthocyanin biosynthesis and promoted its accumulation [7]. In addition, post-harvest dehydration could modulate phenylpropanoid metabolism in different grapevine genotypes, increase the accumulation of flavonoids, and improve the quality of wine [8]. However, compared with research on grape quality development during the on-vine stage, the regulatory mechanism of post-harvest technology has not been fully elucidated and warrants further study.

The post-harvest UV-C technique is widely applied in the storage of various fresh fruits and vegetables. This method can not only improve the secondary quality of post-harvest fruit but also provide sufficient microbial inactivation to preserve food, serving as a nonthermal processing technique [9]. UV light treatment combined with chitosan treatment can better maintain the content of anthocyanins and polyphenols in post-harvest sweet cherries and extend their shelf life [10]. Post-harvest UV-C treatment integrated with ethylene treatment increased the content of flavonols in the edible part of onions [11]. In grapes, UV-C treatment can promote the accumulation of secondary metabolites and improve the quality of post-harvest berries [6,12,13]. However, the specific pathway and comprehensive network by which UV-C treatment regulates post-harvest quality development has not been elucidated to date [14]. Grapes can be divided into red and white varieties, and whether UV-C treatment has the same effect on grapes with different genotypes and whether pathways other than flavonoids biosynthesis can also be regulated warrant further research. Therefore, the white variety ‘Xiangfei’ and the red variety ‘Zicui’ were selected as experimental materials, and a combined multimetabolomic and transcriptomic analysis was utilized to explore the short- and long-term effects of post-harvest UV-C irradiation to establish a theoretical foundation for further research investigating UV-C techniques to efficiently improve post-harvest grape berry quality.

## 2. Methods

### 2.1. Plant Materials and Treatments

The two grape varieties ‘Xiangfei’ and ‘Zicui’ were collected from the vineyard of Northwest A&F University in Yangling, Shaanxi Province, China. According to the commercial maturity characteristics of the variety, grape berries were collected when the total soluble solids (TSS) content and titratable acid (TA) content of ‘Xiangfei’ berries reached 16 °Brix and 6 g L^−1^, respectively, and those of ‘Zicui’ reached 18 °Brix and 4 g L^−1^, respectively. A total of 50 bunches of each variety were collected, and berries with poor maturity were removed.

To accurately evaluate the effect of UV-C on the endogenous metabolism of post-harvest grape berries, the experiment was performed in a culture room (20 °C, RH 80–90%). The control group (8 bunches of each variety per replicate, 3 replicates) was left in darkness, whereas the experimental group (8 bunches of each variety per replicate, 3 replicates) was treated with UV-C lights (TUV 15 W, Philips, Amsterdam, The Netherlands). A UV-C digital radiometer (LH-126C, Lianhui Co., Ltd., Guangdong, China) was used to measure the light intensity, and the lamp position was adjusted until the radiation intensity received on the grape surface reached 18.75 mW m^−2^. The entire bunch surface was exposed to UV-C irradiation and the UV dose was calculated as the product of light intensity and actual exposure time. The long-term treated samples (8 d) received a radiation dose of 3.6 KJ m^−2^ and the short-term treated samples (1 d) received a radiation dose of 0.45 KJ m^−2^. 

According to our preliminary test, the skins of ‘Xiangfei’ berries began to lose commercial value when the continuous treatment of UV-C exceeded 9 days. The samples under continuous UV-C radiation and continuous darkness on the 1st and 8th day were collected for RNA sequencing and the detection of metabolic components to evaluate the effect of short- and long-term UV-C irradiation. A total of 240 berries per treatment (80 per replicate, 10 per bunch) randomly taken from the treated bunches were collected for further different analysis.

### 2.2. Determination of Physiological Indexes of Grape Berries

The contents of TSS and TA were determined according to the method of Azuma et al. [7]. The TSS content was measured using a portable refractometer (PAL-1, Atago, Japan), and the TA content was titrated with 0.1 mol L^−1^ NaOH. The acidity was expressed as the mass of tartaric acid (g L^−1^). MDA was extracted with 10% trichloroacetic acid (TCA) and determined by the thiobarbituric acid (TBA) method [15]. The proline content was determined by the acid-ninhydrin method [16].

### 2.3. Metabolome Detection and Analysis

The grape samples were freeze-dried in a vacuum and were analyzed by UHPLC-ESI-MS, which was performed using a Vanquish UHPLC system (Thermo Fisher, Frankfurt, Germany) coupled with an Orbitrap Q ExactiveTMHF-X mass spectrometer (Thermo Fisher, Germany). A Hypersil Gold column (100 × 2.1 mm, 1.9 μm) was used to separate metabolic components from the sample. The eluents for the positive polarity mode were eluent A (0.1% FA in water) and eluent B (methanol), and those for the negative polarity mode were eluent A (5 mM ammonium acetate, pH 9.0) and eluent B (methanol). The positive and negative elution (eluent A: eluent B) gradient was as follows: 98:2 *v*/*v* at 0 min, 0:100 *v*/*v* at 1.5 min, 0:100 *v*/*v* at 12.0 min, 0:100 *v*/*v* at 14.0 min, 98:2 *v*/*v* at 14.1 min, and 98:2 *v*/*v* at 17.0 min. A QExactiveTMHF-X mass spectrometer was operated in positive/negative polarity mode with a capillary temperature of 320 °C, spray voltage of 3.2 kV, aux gas flow rate of 10 arb, and sheath gas flow rate of 40 arb.

Compound Discoverer 3.1 (CD3.1, Thermo Fisher) was utilized to process the raw data files generated by UHPLC-MS/MS to perform peak picking, peak alignment, and quantitation for each metabolite. By comparing the retention time, m/z, and ion peak mode of the metabolites with those in the standard database, the mzCloud (https://www.mzcloud.org/, accessed on 17 August 2020), and the MassBank databases (www.massbank.jp, accessed on 19 August 2020), the quality of metabolic components was determined. The peak area was used for quantitative analysis. The quality control (QC) sample was set to evaluate the system stability during the experiment, and the blank sample was used to remove background ions. The statistical software R (R version R-3.4.3), Python (Python 2.7.6 version), and CentOS (CentOS release 6.6) were used for statistical analysis.

### 2.4. ESI-Q TRAP-MS/MS for Targeted Flavonoids Component Detection and Analysis

A total of 100 milligrams of grape samples that had been freeze-dried in a vacuum was dissolved in 1 mL of extraction solution (70% methanol/water, *v*/*v*). After vortexing at 4 °C, centrifugation (10,000× *g*, 10 min), and filtration (0.22-μm pore size), the sample extracts were analyzed using an LC-ESI-MS/MS system (HPLC, Shim-pack UFLC SHIMADZU CBM30A system, www. shimadzu.com.cn/; MS, Applied Biosystems 4500 Q TRAP, www.appliedbiosystems.com.cn/). Waters ACQUITY UPLC HSS T3 C18 (1.8 µm, 2.1 mm * 100 mm) was used as the HPLC column. The water phase (eluent A) was ultrapure water (0.04% acetic acid added), and the organic phase (eluent B) was acetonitrile (0.04% acetic acid added). The analysis conditions were as follows: Flow rate at 0.4 mL/min; column temperature 40 °C and injection volume at 5 μL. The elution (eluent A: eluent B) gradient was as follows: 100:0 *v*/*v* at 0 min, 5:95 *v*/*v* at 11.0 min, 5:95 *v*/*v* at 12.0 min, 95:5 *v*/*v* at 12.1 min, and 95:5 *v*/*v* at 15.0 min. The effluent was subsequently connected to an ESI-triple quadrupole-linear ion trap (Q TRAP)-API 4500 Q TRAP LC/MS/MS System, on which LIT and triple quadrupole (QQQ) scans were acquired. Polypropylene glycol solutions (10 and 100 μmol/L) were employed for instrument tuning and mass calibration in QQQ and LIT modes, respectively. In QQQ, each ion pair was scanned based on the optimized declustering potential (DP) and collision energy (CE) [17]. Based on the local self-built database MWDB (Metware database, Metware Biotechnology Co., Ltd., Wuhan, China) of flavonoids, and qualitative and quantitative analyses of the metabolites were performed.

### 2.5. Transcriptomic Analysis

#### 2.5.1. RNA Library Construction and Sequencing

RNA extraction and integrity testing were performed according to the method of Zhang et al. [18]. The RNA Nano 6000 Assay Kit of the Bioanalyzer 2100 system (Agilent Technologies, Santa Clara, CA, USA) was used to test the integrity of the extracted RNA. The total RNA qualified for the integrity test was subjected to the steps of enrichment, random interruption, reverse transcription amplification, and purification to generate double-stranded cDNA. Then PCR was performed with Phusion High-Fidelity DNA polymerase, universal PCR primers, and Index (X) Primer. Finally, PCR products were purified (AMPure XP system), and library quality was assessed on the Agilent Bioanalyzer 2100 system. After clustering of the index-coded samples, the library preparations were sequenced on an Illumina NovaSeq platform, and 150-bp paired-end reads were generated.

#### 2.5.2. Data Quality Control, Sequence Alignment, and New Transcript Prediction

Clean data were acquired when the reads with adapters, N (N means base information cannot be determined), and low quality (Qphred < 20 bases account for more than 50% of the total read length) were removed from raw data. After analysis of Q20, Q30, and GC(Cytosine and Guanine) content calculation, clean data of high quality were utilized for subsequent analysis.

The reference genome was downloaded from the database EnsemblPlants (http://plants.ensembl.org/info/data/ftp/index.html, accessed on 19 August 2020), and HISAT2v2.0.5 was used to construct the index of the reference genome and perform sequence alignment [19]. New genes were predicted based on StringTie (1.3.3b) [20].

#### 2.5.3. Quantification of Gene Transcription Levels and Analysis of DEGs

Based on featureCounts (1.5.0-p3), the read numbers mapped to each gene were calculated [21]. Next, the FPKM (expected number of fragments per kilobase of transcript sequence per million base pairs sequenced) of each gene was calculated according to the length of the gene and read numbers mapped to the gene. The DESeq2 R package (1.16.1) was used to analyze the differentially expressed genes between the two comparison groups. The *p*-value was adjusted to control the false discovery rate based on the method of Benjamini and Hochberg [22]. Genes with |log2foldchange|>0 and the corrected *p*-values < 0.05 were determined to be DEGs.

#### 2.5.4. Pathways Enrichment Analysis of DEGs

The Gene Ontology (GO) resource provides a computational representation of current scientific knowledge regarding the functions of genes, including how individual genes contribute to the biology of an organism at the molecular, cellular, and organism levels. Gene Ontology enrichment analysis of DEGs was performed using clusterProfiler (3.4.4) software, and GO terms with corrected *p*-values < 0.05 were enriched. 

The Kyoto Encyclopedia of Genes and Genomes (KEGG, http://www.genome.jp/kegg/, accessed on 15 September 2020) is an efficient database resource for analyzing genome sequencing data and gene functions. The integrated database resource consists of 18 databases, which are broadly categorized into systems information, genomic information, chemical information, and health information. ClusterProfiler (3.4.4) was also used to analyze the DEG enrichment in KEGG pathways.

### 2.6. qRT-PCR Analysis

Total RNA isolation, cDNA synthesis, and gene expression analysis were carried out according to our previously described method [23]. The 13 differentially expressed genes shared by each comparison group were screened out for qRT-PCR verification of expression levels. Primer Premier 6.0 was utilized for primer design (Table S3), and *ubiquitin* and *EF1γ* were utilized as internal standards for expression normalization. qRT-PCR was performed using the CFX96 Real-Time PCR Detection system (Bio-Rad, Hercules, CA, USA), and expression levels were calculated based on the 2^-△△Ct^ method.

## 3. Results

### 3.1. Effect of UV-C Treatment on the Appearance and Physiological Indexes of Grape Berries

**Figure 1:**
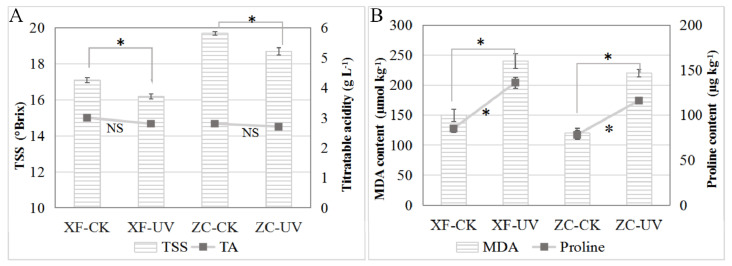
Effect of post-harvest UV-C application on the content of total soluble solids (TSS), titratable acid (TA), malondialdehyde (MDA), and proline in grape berries. (**A**) Comparison of TSS and TA contents in ‘Xiangfei’ and ‘Zicui’ berries under different treatments. The bar graph represents the difference in TSS content, and the line graph represents the difference in TA content. The error bars represent standard deviations of the means. The asterisk indicates significant differences (*p* < 0.05, *t*-tests), while NS means that the difference is not significant. XF represents ‘Xiangfei’ and ZC represents ‘Zicui’. CK represents the control dark treatment while UV represents the UV-C treatment. The same below follows. (**B**) Comparison of MDA and proline contents in ‘Xiangfei’ and ‘Zicui’ berries under different treatments. The bar graph represents the difference in MDA content, and the line graph represents the difference in proline content.

By comparing the TSS and TA contents in the berries stored on the 8th day (Figure 1), it was observed that the TSS content in the berries of ‘Xiangfei’ and ‘Zicui’ under UV-C treatment decreased significantly, while the titratable acid content changed only slightly. Additionally, it was also observed that UV-C treatment could increase the content of MDA and proline in the two varieties (Figure 1B), indicating that UV-C triggered stress responses in the berries. Meanwhile, after long-term UV-C treatment, the red variety ‘Zicui’ showed a more intense purple-black color, and the white variety ‘Xiangfei’ appeared light yellow-brown (Figure S1), indicating that UV-C affected the accumulation of coloring-related metabolites.

### 3.2. Effect of UV-C on Gene Transcription Abundance in Post-Harvest Grape Berries

#### General Description of Transcriptomic Data

We obtained transcriptomic data of 24 samples in total, which were divided into eight thesis (‘Xiangfei’ under the control treatment on the 1st day, ‘Xiangfei’ under the UV-C treatment on the 1st day, ‘Zicui’ under control on the 1st day, ‘Zicui’ under UV-C on the 1st day, ‘Xiangfei’ under control on the 8th day, ‘Xiangfei’ under UV-C on the 8th day, ‘Zicui’ under control on the 8th day, and ‘Zicui’ under UV-C on the 8th day) with three replicates each. An average of 6.28 G data and 41,865,788 clean reads were obtained for each sample (Table S1). Compared with the grape genome database, the mapping rate of the samples was 87.6%, fully reflecting the changes in the transcription level of grape berries under different treatment conditions. Meanwhile, 1066 new genes were identified in this study (Table S2), which may be employed in further functional research.

### 3.3. DEGs Analysis between Different Treatments

**Figure 2:**
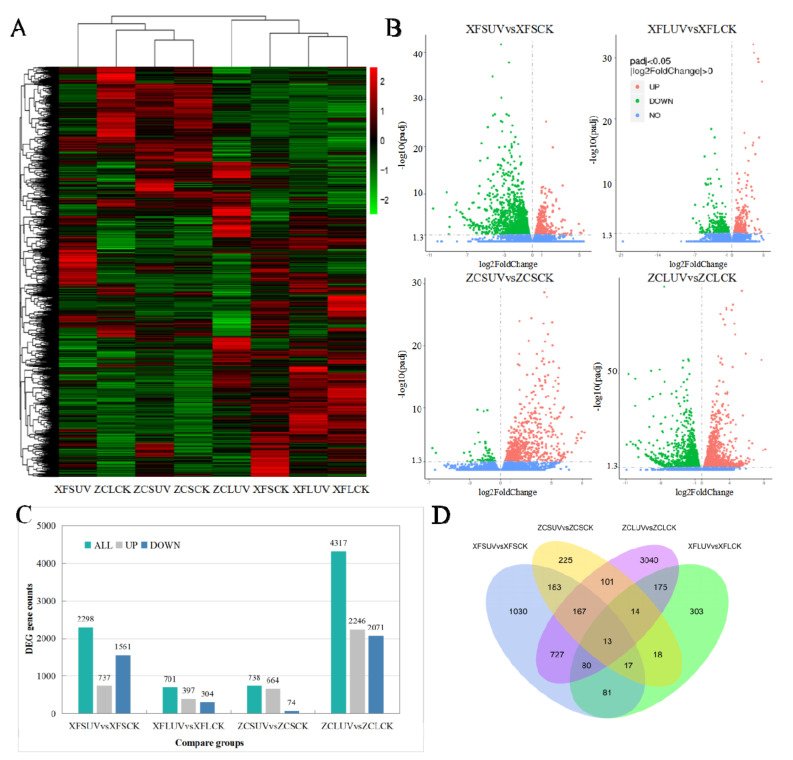
Differentially expressed genes (DEGs) of different compare groups. (**A**) Comparison of the transcription level of DEGs. (**B**) Volcano map for the significance level of the DEGs. The horizontal axis represents the fold change of DEGs, and the vertical axis represents the significance level of the difference. Padj indicates the corrected *p* value after the multiple hypothesis test, the same as below. (**C**) Comparison of the DEG numbers in different comparison groups. (**D**) The number of shared or specific DEGs between different comparison groups. XFSUV/XFSCK indicates ‘Xiangfei’ under short-term UV-C/control conditions. XFLUV/XFLCK indicates ‘Xiangfei’ under long-term UV-C/control conditions. ZCSUV/ZCSCK indicates ‘Zicui’ under short-term UV-C/control conditions. ZCLUV/ZCLCK means ‘Zicui’ under long-term UV-C/control condition vs. means the change of the former relative to the latter. The same as below.

DEGs were primarily analyzed between UV-C-treated samples and control samples. The comparison group names were listed as XFSUV vs. XFSCK, XFLUV vs. XFLCK, ZCSUV vs. ZCSCK, and ZCLUV vs. ZCLCK. XFSUV/XFSCK indicates ‘Xiangfei’ under short-term UV-C/control conditions. XFLUV/XFLCK indicates ‘Xiangfei’ under long-term UV-C/control conditions. ZCSUV/ZCSCK indicates ‘Zicui’ under short-term UV-C/control conditions. ZCLUV/ZCLCK means ‘Zicui’ under long-term UV-C/control condition. The transcription levels of DEGs between different treatment groups are presented in the cluster heat map of Figure 2A. Based on the comparison of gene expression levels, all samples were divided into two groups, but the two groups were not entirely classified according to the variety or radiation conditions, suggesting that the responses of different varieties to UV-C have both common characteristics and specific properties. UV-C also induced a difference in the number of DEGs (Figure 2B,C). The number of DEGs in ‘Zicui’ samples exposed to long-term UV-C irradiation was the largest, while that in ‘Xiangfei’ samples under the same treatment was the smallest. With the extension of UV-C treatment time, the number of DEGs in ‘Zicui’ further increased, whereas that in ‘Xiangfei’ further decreased.

The number of upregulated DEGs in the berries under UV-C treatment was greater than the number of downregulated genes, except for ‘Xiangfei’ grapes under short-term irradiation (XFSUV vs. XFSCK). The number of common and specific DEGs between different comparison groups is shown in the Venn diagram (Figure 2D). There were 191 genes in ‘Xiangfei’ and 295 genes in ‘Zicui’ that were sensitive to both short- and long-term UV-C irradiation. After short-term UV-C treatment, the transcription levels of 380 genes changed simultaneously in ‘Xiangfei’ and ‘Zicui’ berries, while after long-term treatment, 282 genes changed. The abovementioned results also indicated that the effect of UV-C on gene transcription was affected by both the UV-C duration and the grapevine genotype. The four comparison groups shared 13 differentially expressed genes. The relative expression levels of these genes, as determined by qRT-PCR, were in keeping with the data obtained in RNA sequencing (Figure S2).

### 3.4. KEGG Pathway and GO Enrichment Analysis of DEGs

**Figure 3:**
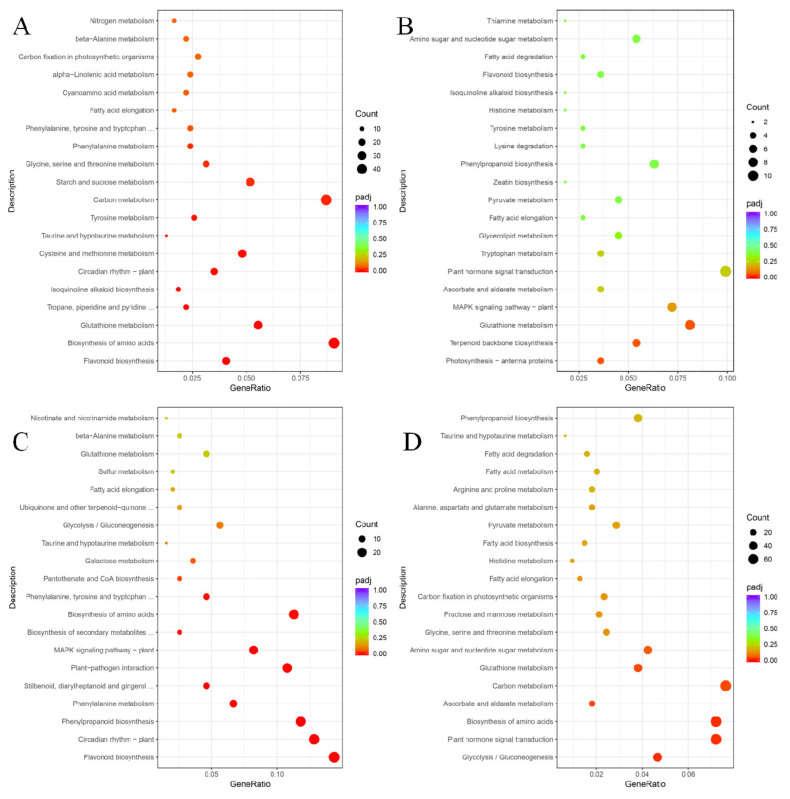
KEGG enrichment pathways of DEGs in different comparison groups. The size of the dot represents the number of differential genes enriched in different pathways, and their color represents the significance level of the difference. The gene ratio represents the ratio of the number of DEGs annotated to the KEGG pathway to the total number of DEGs. (**A**, **B**, **C**, and **D**) represent the comparison group of XFSUV vs. XFSCK, XFLUV vs. XFLCK, ZCSUV vs. ZCSCK, and ZCLUV vs. ZCLCK, respectively.

To better describe the differences in metabolic pathways responding to UV-C in post-harvest grape berries, the KEGG pathway and GO enrichment analyses of DEGs were used. Molecular networks are the most unique data object in KEGG, which construct molecular interaction, reaction and relation networks, and represent systemic functions of the cell and organism [24]. In the KEGG database, we screened the top 20 differential metabolic pathways with higher correlation coefficients according to the padj value (the smaller the padj value is, the higher the correlation is) for comparative analysis (Figure 3).

Glutathione metabolism and fatty acid elongation were enriched with DEGs in the metabolic pathway maps of all four comparison groups. Except for the ‘Zicui’ berries under long-term UV-C treatment (including 15 differential genes, although its significance value did not reach the threshold), the flavonoid biosynthesis pathway appeared in all other treatments. The phenylpropanoid biosynthesis pathway was enriched in the XFLUV vs. XFLCK, ZCSUV vs. ZCSCK, and ZCLUV vs. ZCLCK groups, while biosynthesis of amino acids and taurine and hypotaurine metabolism were enriched in all groups except XFLUV vs. XFLCK. The above results reflected that the metabolic processes of glutathione metabolism, taurine and hypotaurine metabolism, fatty acid elongation, flavonoid biosynthesis, phenylpropanoid biosynthesis, and biosynthesis of amino acids in the two varieties were all able to respond to UV-C signals.

From the perspective of genotypes, glycolysis/gluconeogenesis was enriched only in UV-C-treated ‘Zicui’ berries, whereas isoquinoline alkaloid biosynthesis and tyrosine metabolism were enriched only in UV-C-treated ‘Xiangfei’ berries. Regarding irradiation duration, circadian rhythm-plant, phenylalanine metabolism, phenylalanine, tyrosine and tryptophan biosynthesis, and beta-alanine metabolism were enriched only in short-term-treated berries, while ascorbate and aldarate metabolism, plant hormone signal transduction, pyruvate metabolism, histidine metabolism, fatty acid degradation, and amino sugar and nucleotide sugar metabolism were enriched only in long-term-treated berries (Figure 3).

**Figure 4:**
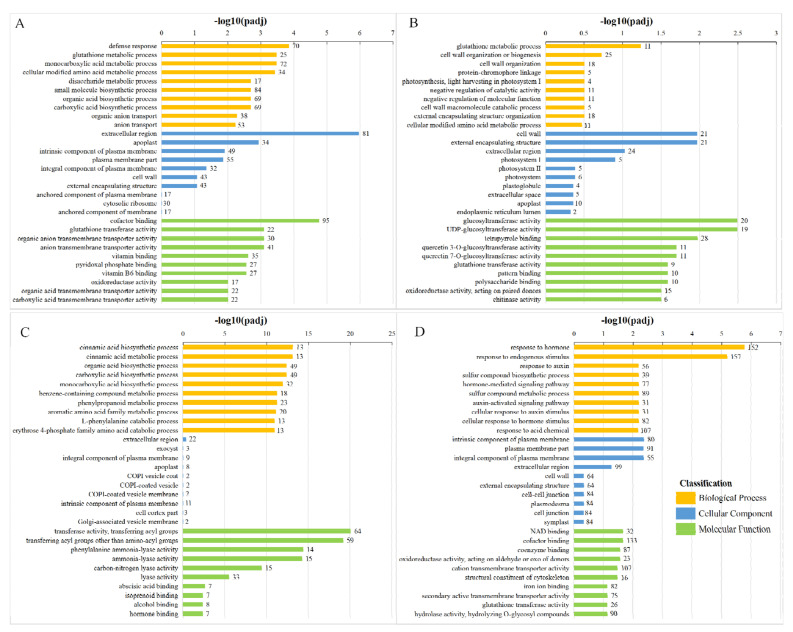
Gene Ontology (GO) enrichment items in different comparison groups. The horizontal axis represents the significance level of the difference. The number next to each bar represents the number of enriched DEGs. (**A**, **B**, **C**, and **D**) represent the comparison group of XFSUV vs XFSCK, XFLUV vs XFLCK, ZCSUV vs ZCSCK, ZCLUV vs ZCLCK, respectively.

The GO database describes the function of genes with respect to the molecular function, cellular component, and biological process. Molecular function terms describe activities that occur at the molecular level. Cellular component describes the locations relative to cellular structures in which a gene product performs a function. Biological processes are biological programs, and larger processes are usually mediated by multiple molecular activities. We screened the top 10 enriched GO terms from each category of the molecular function, cellular component, and biological process (Figure 4). 

Except for the XFLUV vs XFLCK group, the enriched pathways in the comparison groups were primarily concentrated in the biological process category. In the XFSUV vs XFSCK group, defence response, extracellular region and cofactor binding were the items with the highest significance and the most enriched genes among the three GO enrichment types, while in the XFLUV vs XFLCK group, cell wall organization or biogenesis, extracellular region, and tetrapyrrole binding were the most enriched items of DEGs in each type. In the ZCSUV vs ZCSCK group, transferase activity, transferring acyl group items, was enriched with 64 differentially expressed genes; this group exhibited the largest number, as well as the highest significance. In the ZCLUV-ZCLCK group, DEGs were more enriched for hormone stimulus, and hormone response-related items in the biological process category, while at the molecular function level, the number of DEGs enriched in cofactor binding items was the largest. In general, with the prolonging of UV-C treatment time, the number of DEGs enriched in various types of GO categories in ‘Xiangfei’ samples showed an overall decreasing trend, while that in ‘Zicui’ samples showed an increasing trend.

### 3.5. Multiple Regulation Pathways Responding to UV-C

**Figure 5:**
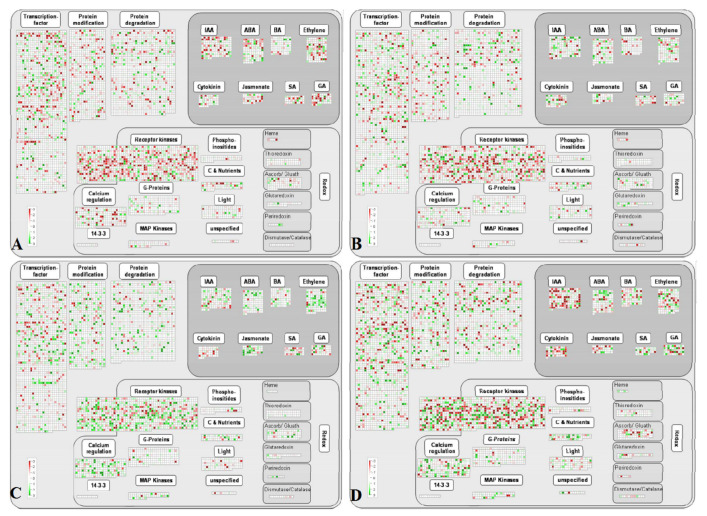
DEGs assigned to the overview of regulatory pathways in different comparison groups based on MapMan. The overview mainly consists of transcription factors, protein modifications, protein degradation, hormone synthesis and signal transduction, redox reactions, receptor kinases, phosphoinositides, C&Nutrients, light, G-proteins, MAP kinases, calcium regulation, and the unspecified part. The relative transcription levels of genes related to each part are displayed in the heatmap. Green represents upregulation, while red represents downregulation. (**A**, **B**, **C**, and **D**) represent the comparison group of XFSUV vs. XFSCK, XFLUV vs. XFLCK, ZCSUV vs. ZCSCK, and ZCLUV vs. ZCLCK, respectively.

UV-C can stimulate the defense system of plants [25]. Various signaling pathways, such as gene transcription regulation, protein modification and degradation, hormone signal transduction, and redox regulation, in post-harvest grape berries were induced by UV-C radiation (Figure 5).

Under short-term UV-C treatment, genes in the thioredoxin, ascorbate/glutathione, glutaredoxin, and peroxiredoxin pathways related to the regulation of ROS homeostasis were activated in both varieties, whereas the transcription levels of most genes in the salicylic acid (SA) biosynthesis pathway were downregulated. In ‘Zicui’ berries, UV-C treatment activated the expression of most genes in the ABA, ethylene, and jasmonate biosynthesis pathways, while in ‘Xiangfei’ berries, only some related genes were activated. The transcription of most genes related to receptor kinases and protein modification was inhibited in ‘Xiangfei’ berries but was upregulated in ‘Zicui’ berries. After long-term UV-C treatment, the transcription of most genes related to IAA and jasmonate biosynthesis and thioredoxin, ascorbate/glutathione, glutaredoxin, and peroxiredoxin in the ROS homeostasis pathway were downregulated, while some genes in the SA pathway were upregulated in ‘Zicui’ berries. In ‘Xiangfei’ berries, the transcription of most genes in IAA and jasmonate biosynthesis pathway did not exhibit a clear downward trend, in contrast to that in ‘Zicui’, and ascorbate/glutathione and glutaredoxin were still upregulated.

With longer periods of UV-C radiation, the biosynthesis pathways of ABA, ethylene, jasmonate, and IAA in ‘Xiangfei’ gradually changed from inhibition to activation, and the antioxidant metabolism-related ascorbate/glutathione and glutaredoxin pathways remained upregulated. However, the transcription of genes related to receptor kinases, protein modification, IAA, ethylene, jasmonate, and ascorbate/glutathione in ‘Zicui’ berries changed from upregulation to downregulation. The above results indicated that hormone biosynthesis- and ROS homeostasis-related signaling pathways might play important roles in UV-C signal transduction, but the time points at which different varieties responded to UV-C signaling and stimulated transcription regulation were probably different.

**Figure 6:**
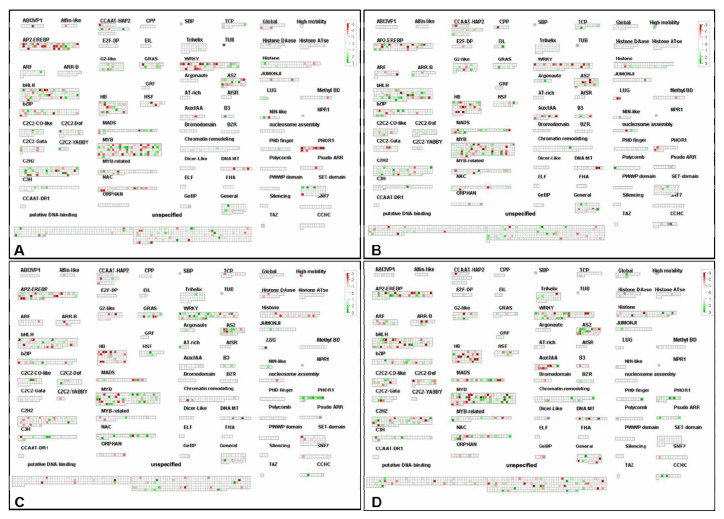
Relative transcription level of genes in different transcription factors. The main transcription factor families are listed in MapMan. Green represents upregulation, while red represents downregulation. (**A**, **B**, **C**, and **D**) represent the comparison group of XFSUV vs. XFSCK, XFLUV vs. XFLCK, ZCSUV vs. ZCSCK, and ZCLUV vs. ZCLCK, respectively.

After UV-C irradiation, the expression levels of various transcription factor family members in post-harvest grape berries were differentially regulated (Figure 6). Under short-term UV-C irradiation, the expression of most WRKY, PHOR1, and NAC gene family members in ‘Xiangfei’ was downregulated, while they were upregulated in ‘Zicui’. After long-term UV-C irradiation, WRKY, PHOR1, and NAC family genes were downregulated in ‘Xiangfei’. The expression levels of HB and AuxIAA family members were also downregulated in the two varieties. Previous studies have determined that UV radiation could function via the basic leucine zipper domain (bZIP) and R2R3-MYB transcription factor family members [26]. In this study, the expression levels of bZIP and MYB family members were upregulated or inhibited in both varieties, while the transcription levels of WRKY, PHOR1, and NAC family genes showed relatively consistent and significant changes after UV-C radiation treatment, indicating that these genes may also play important roles in the signal transduction process.

### 3.6. Metabolomic Analysis

#### 3.6.1. Analysis of Differentially Accumulated Metabolites

The metabolites in different samples were detected by UHPLC-ESI-MS. After removing the substances with low peak values and low similarity by setting the reference threshold, we identified a total of 1483 compounds (Table S4) in grape berries in positive mode and 680 compounds in negative mode.

**Figure 7:**
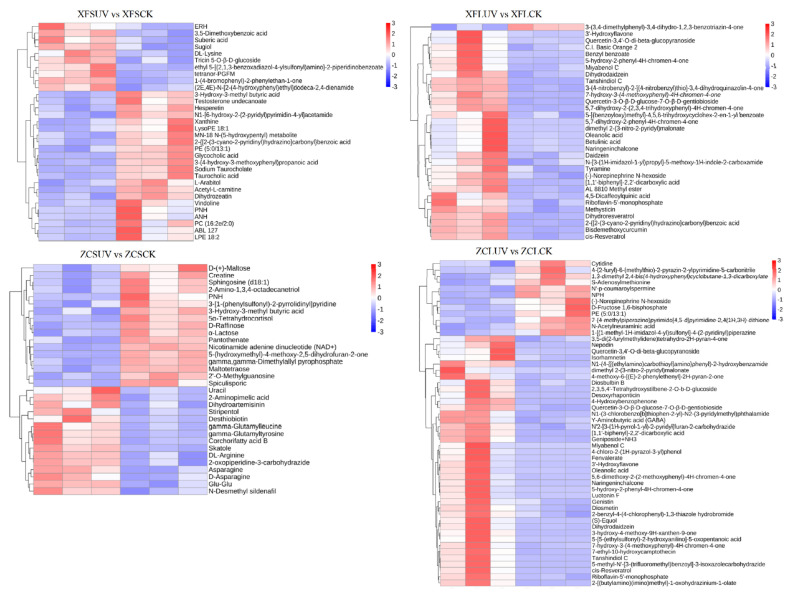
Heatmap of the relative content of differential metabolites between different groups in positive mode. The first three columns were the three replicates of the experimental group (UV-C), and the last three columns were that of the control group. Red represents upregulation, while blue represents downregulation.

The screening of the differentially accumulated metabolites between different samples was primarily dependent on VIP (variable importance in the projection), FC (fold change), and *p*-value. VIP refers to the variable projection importance of the first principal component in the PLS-DA model [27], representing the contribution of metabolites to the group classification. FC is the ratio of the quantitative mean of each metabolite in the comparison group. The *p*-value was calculated by a t-test, indicating the significance level of the difference. In this experiment, the threshold was set as VIP > 1.0, FC > 1.5, or FC < 0.667 and *p*-value < 0.05 [27,28]. In the different comparison groups, the types and contents of differential metabolites were not completely identical (Figure 7). In general, after long-term UV-C treatment, the number of substances with increased content and decreased content in berries were more and less than that of samples treated with short-term UV-C, respectively. A total of 49 (32/17 in pos/neg), 51 (32/19 in pos/neg), 39 (33/6 in pos/neg), and 84 (52/32 in pos/neg) differential compounds in XFSUV vs. XFSCK, XFLUV vs. XFLCK, ZCSUV vs. ZCSCK, and ZCLUV vs. ZCLCK groups, were listed, respectively (Figure 7, Figure S3). Resveratrol, as a nonflavonoid polyphenol compound, has attracted widespread attention for its strong antioxidant and health benefits. After long-term UV-C treatment, the content of cis-resveratrol in the berries of the two varieties increased significantly.

The enrichment pathway of differentially accumulated metabolites between the comparison groups was also affected by the variety and UV-C treatment time. In addition to the ZCSUV vs. ZCSCK group, flavonoid biosynthesis and biosynthesis of secondary metabolite pathways were widely present in each comparison group (Figure S4), indicating that regardless of the variety, the flavonoids and secondary metabolite synthesis pathways could respond to the UV-C treatment.

#### 3.6.2. Analysis of Differentially Accumulated Metabolites in the Targeted Metabolite of Flavonoids

**Figure 8:**
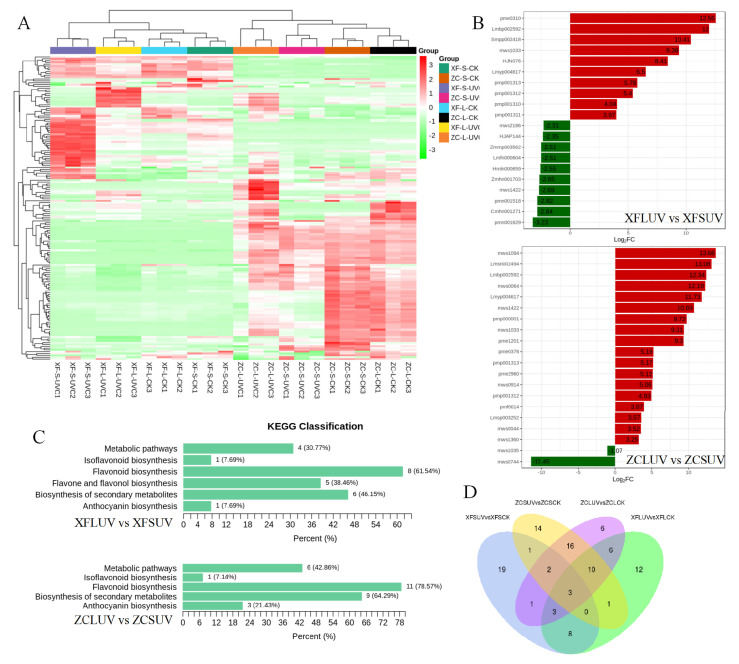
Characteristics of flavonoids accumulation in grape berries under UV-C treatment. (**A**) Cluster heat map of flavonoids in each sample. Red represents upregulation, while green represents downregulation. (**B**) Top fold change metabolites in the comparison group of XFLUV vs. XFSUV and ZCLUV vs. ZCSUV. Components in the top 20 Log_2_FC are listed, and their information is provided in Table S6. (**C**) KEGG enrichment of differential metabolites in the comparison group of XFLUV vs. XFSUV and ZCLUV vs. ZCSUV. The number next to each bar represents the number of enriched differential metabolites, and cluster_frequency in brackets represents the ratio of the number of differential metabolites to the total number of differential metabolites annotated in KEGG. (**D**) The intersection of differential flavonoids between different treatment groups.

To further analyze the effect of UV-C on flavonoids, targeted metabolome analysis of flavonoids was applied in all 24 samples. A total of 165 flavonoids, including six chalcones, seven dihydroflavones, eight dihydroflavonols, 21 anthocyanins, 38 flavonoid, 45 flavonols, five flavonoid carbonosides, 10 flavanols, 15 tannins, and 10 proanthocyanidins, were detected (Table S5). When naming the components, ‘flavonoids’ represents all subgroups, but ‘flavonoid’ just represents one subgroup of them. The effect of post-harvest UV-C application on the accumulation of flavonoids was also dependent on the grapevine genotypes and irradiation duration (Figure 8A). Under short-term UV-C treatment, the contents of almost all tannin, proanthocyanidin, chalcone, and flavanol components and some flavonoid subgroup components in the differential metabolites (XFSUV vs. XFSCK) exhibited an increasing trend in ‘Xiangfei’ berries (Table S6), while the contents of all chalcone, dihydroflavone, flavonoid, flavanol, and tannin components also showed an upward trend under long-term UV-C treatment (XFLUV-XFLCK). In the ‘Zicui’ berries, the contents of anthocyanins and proanthocyanidins changed insignificantly, but the contents of flavonoid and flavonols showed a downward trend under short-term UV-C treatment. Under the long-term UV-C treatment, the contents of some flavonols and anthocyanins showed an upward trend.

**Figure 9:**
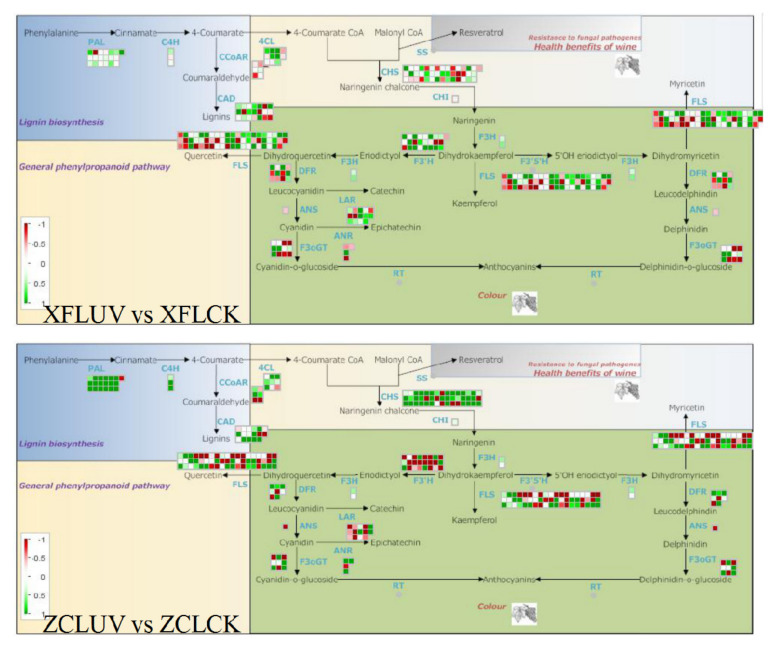
Transcription level of structural genes involved in the general phenylpropanoid pathway based on MapMan. Green represents upregulation, while red represents downregulation.

With the extension of UV-C treatment time, the content of ethyl gallate (pme0310), kaempferol-3,7-di-O-glucoside (Lmbp002592) and petunidin-3-O-(6’’-O-acetyl)glucoside (Smpp002418) in the ‘Xiangfei’ berries increased by more than 10 times (fold change). Aromadendrin (mws1094), ellagic acid-4-O-xyloside (Lmsn002494), kaempferol-3,7-di-O-glucoside (Lmbp002592), eriodictyol (mws0064), pinocembrin-7-O-glucoside (Lmyp004617), and epiafzelechin (mws1422) contents showed a significant increase in ‘Zicui’ berries (Figure 8B). By KEGG classification analysis, most differential metabolites were enriched in the flavonoid biosynthesis pathway in the two varieties (Figure 8C). After long-term UV-C treatment, the expression levels of most members of the flavonoid biosynthesis-related *PAL*, *C4H*, and *4CL* genes in the two varieties were upregulated (Figure 9), while the expression levels of other synthetic genes were regulated differently, reflecting the effect of genotype on the phenylpropanoid pathway responding to UV-C. Meanwhile, the expression level of some members of the *VvSS* (*VvSTS*) family, key genes involved in the biosynthesis of resveratrol, was also upregulated, consistent with the accumulation of cis-resveratrol in the berries. UV-C can affect the biosynthesis of flavonoids and cis-resveratrol by regulating the expression level of the phenylpropanoid pathway. 

## 4. Discussion

Post-harvest fruit still exhibits biological activities, for which many metabolic processes involved in the accumulation of endogenous metabolites, cell development, and stress response can be affected by post-harvest technology. When post-harvest grape berries undergo dehydration or commence senescence, various metabolic pathways involved in the activity of enzymes related to anaerobic respiration and the structure of cell wall polysaccharides gradually change [29,30]. Hormone regulation pathways in the berries, such as genes in auxin and ethylene metabolism, and stress response pathways, such as defense responses and oxidative and osmotic stress, are consistently modulated [8]. The grape quality of the two varieties in this study was regulated by the application of post-harvest UV-C. The soluble solids content in the berries decreased, while the content of secondary metabolites, such as flavonoids and resveratrol, increased. Solids may be transformed under UV-C stimulation. Biosynthesis of secondary metabolites, hormone signal transduction pathways and stress response pathways can all respond to UV-C irradiation. Integrating those transcriptome and metabolic profile data, it was revealed that UV-C activated the stress response in grape berries, ROS homeostasis and hormone synthesis pathways may be involved in the transduction of stress signals, and the secondary metabolites biosynthesis pathways were finally activated. The accumulated flavonoids and resveratrol may be used to protect grape berries from stress damage. 

Post-harvest techniques involved in the process of handling, storage, packing, and shipping are mainly used to extend shelf life and maintain and improve the post-harvest quality of fresh vegetables and fruit [31]. The evaluation of the effects of ultraviolet (UV) irradiation has been extensively studied [14]. Visible light affects the biosynthesis of proanthocyanins, while UV radiation regulates the biosynthesis of flavonols [3,32]. The effects of UV-C on fruit and juices are currently under study. UV-C treatment is a nonthermal processing technique for the preservation of food [9], which can cause inactivation of spoilage yeast in grape must without causing off-flavor formation in wines. The duration of the dark period following UV-C exposure was also found to affect the control of *Botrytis cinerea* [33]. Under UV-C irradiation, the stilbene content increases in post-harvest grape berries, and the final wine is enriched approximately 2- and 1.5-fold in resveratrol and piceatannol, respectively [34]. The total phenolic compound, flavonoid, flavanol, and anthocyanin contents and antioxidant activities of grapes after UV-C treatments were higher than other treatments, and the expression level of phenolic component biosynthesis genes was the highest under UV-C radiation [6]. Stilbenoids and isoprenoids (such as terpenes and carotenoids) might function as one part of the UV-response machinery in grape berries [26,35]. Similar to previous studies, the transcription level of many genes involved in the biosynthesis of flavonoids and resveratrol, such as *VvPAL*, *VvC4H*, *Vv4CL*, *VvCHS*, *VvSS(VvSTS)*, *VvF3H*, *VvDFR*, and *VvLAR* were up-regulated under the irradiation of UV-C (Figure 9) and many flavonoids and cis-resveratrol accumulated accordingly in the berries. Meanwhile, it was also found that with the extension of UV-C irradiation, flavonols and anthocyanins mainly accumulated in the red variety ‘‘Zicui’’, whereas flavonoid, flavanol, and tannin components mainly accumulated in the white variety of ‘‘Xiangfei’’. These accumulated flavonoids modulate the coloring properties of grape berries. For the red grape, “Zicui”, the activation of the anthocyanin biosynthesis pathway and the accumulation of anthocyanins make the berries appear more intense purple-black and improve the organoleptic quality of the grape berries. More importantly, due to the high antioxidant activity of flavonoids and their positive effect on maintaining human health [1,2], the accumulation of flavonoids and cis-resveratrol induced by UV-C irradiation is conducive to improving the nutritional value and commerciality of grape berries. 

Ultraviolet radiation could cause multiple damage to plants. UV-B changed the degree of DNA methylation [36], and UV-C energy reacted photochemically with DNA and caused damage to the DNA double-strand structure [37], which would make it almost impossible for injured cells to carry out DNA replication. Continuous UV-C irradiation also caused an overproduction of reactive oxygen species (ROS) in plants, causing peroxidation of cell membranes, cell structure damage, and functional destruction as well as stimulating the antioxidant enzyme system [13]. In this study, the expression of thioredoxin, ascorbate/glutathione, glutaredoxin, and peroxiredoxin pathway genes related to ROS homeostasis changed after UV-C irradiation, and protective components, such as MDA and proline, accumulated in grape berries, which suggests that under UV-C treatment, the grapes activated their own defense mechanism, and the reaction to remove reactive oxygen species and stabilize the redox state was active [38].

Various hormone signaling pathways also participate in the response to UV-C, in which the SA protection mechanism is involved. PROHIBITIN3 forms a complex with isochorismate synthase 1 to regulate the production of SA in Arabidopsis in response to UV-C stress [25]. The SA signaling pathway also cooperates with JA signaling to regulate the defense response of plants [39]. In this study, the expression of isochorismate (SA biosynthesis precursor) biosynthesis genes (Figure S5) and JA biosynthesis pathway-related genes was upregulated at the initial stage of UV-C irradiation in “Zicui” berries, indicating that the SA and JA signaling pathways also played important roles in the response to UV-C stress. In “Xiangfei” berries, the SA biosynthesis pathway was downregulated under UV-C radiation, whereas the JA signaling pathway gradually changed from the downregulated state to the upregulated state. The JA signaling pathway may play a more important role in the defense response of the white genotype. In addition to the SA and JA pathways, the expression of ABA and ethylene biosynthesis-related genes also changed in “Zicui” berries. ABA and ethylene signaling pathways may also be involved in the transduction of UV-C signals, but the specific mechanism needs further verification.

Plants may increase their tolerance to UV-C stress by regulating the accumulation of flavonoids [40]. According to their structure, flavonoids are mainly divided into flavonols, flavones, isoflavones, and anthocyanins. These substances not only affect the coloring properties of fruit but also function as free radical scavengers to locate and neutralize free radicals to protect cells from damage [41]. Pre-harvest UV-C treatment can increase the expression levels of *FaCHS1*, *FaCHI*, *FaFHT*, *FaDFR*, *FaFLS*, and *FaFGT* to promote the accumulation of polyphenols in strawberry fruit [42]. Stilbene biosynthesis could be promoted by MYB14 under UV-C [43]. UV-B can affect low molecular weight polyphenol (LMWP) accumulation by promoting DNA methylation to regulate the acclimation mechanisms of plants [36]. In this study, UV-C activated the biosynthesis and accumulation of flavonoids, which improved their nutritional value and the tolerance of the berries to stress. However, the effect of UV-C on the accumulation of phenolics might be affected by berry characteristics. UV-C was more conducive to the accumulation of phenolics in conventional grapes but had little effect on the total phenolic content in organic grapes, in which the content of flavonol decreased under UV-C conditions [12]. In this study, UV-C promoted the accumulation of various types of flavonoids in the berries of the two varieties, but the content of some flavonoids (mainly flavonols) in “Zicui” decreased at the initial stage of UV-C irradiation, which may be observed because the original flavonols being used for free radical scavenging and a competition with other flavonoids for precursors.

**Figure 10:**
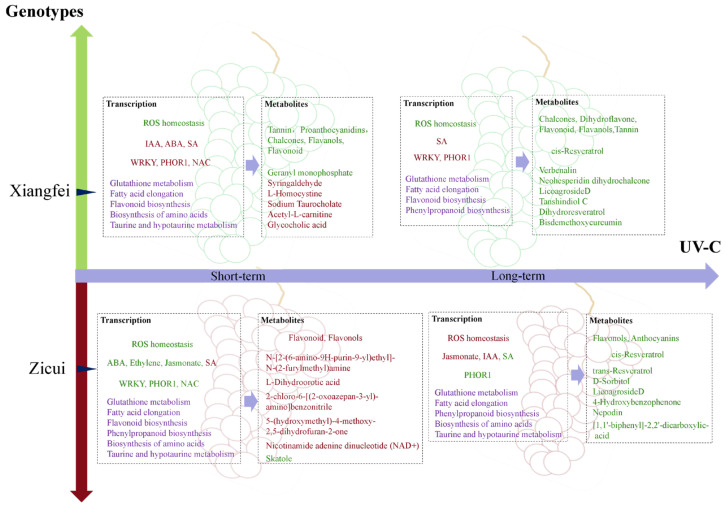
A summary of post-harvest UV-C application regulating the quality development of grape berries. The gene transcription part mainly included ROS homeostasis, hormone synthesis pathway, transcriptional regulatory factors, and quality component-related metabolism. Hormones and transcriptional regulators with obvious changing patterns and the differential metabolic pathways commonly present in the two cultivars after UV-C treatment were all listed in the summary. In the metabolites section, flavonoids and other differential metabolites (the top three most significant metabolites in the pos and neg modes according to the *p*-value) were listed. Green represents the upregulated transcription or accumulation level, while red represents the downregulated transcription or accumulation level. Purple represents a significant difference compared to the control.

Taken together, these results indicate that UV-C could regulate the primary and secondary metabolic processes in post-harvest grapes through the ROS homeostasis system and hormone signaling process. Metabolic pathways related to berry quality traits, such as the biosynthesis of flavonoids and resveratrol, fatty acid elongation, and biosynthesis of amino acids, differ according to variety and UV-C treatment time (Figure 10).

## 5. Conclusions

Both the variety and duration of radiation affected the regulation of gene transcription and metabolite accumulation by UV-C. Compared with the control, the DEGs under UV-C treatment were enriched in pathways related to metabolite accumulation, hormone biosynthesis, and signal transduction, as well as ROS homeostasis. Flavonoid biosynthesis and biosynthesis of secondary metabolites were enriched with differential metabolites. After long-term UV-C irradiation, cis-resveratrol accumulated in the berries of the two varieties, while the differential chalcone, dihydroflavone, flavonoid, flavanol, and tannin components primarily accumulated in “Xiangfei”, and some flavonols and anthocyanins mainly accumulated in “Zicui”. The newly constructed molecular network of post-harvest grape berries responding to UV-C signals may provide theoretical support to further research attempting to comprehensively clarify the regulatory mechanism of UV-C and use it more efficiently to improve post-harvest fruit quality.

## Data Availability

Data is contained within the article or supplementary material.

## Supplementary Materials

The following are available online at https://www.mdpi.com/2304-8158/10/3/625/s1, Figure S1: The effect of UV-C on the appearance of grape berries. Figure S2: Comparison of the gene transcription levels detected by RNA sequencing and qRT-PCR. The 13 differentially expressed genes shared by each comparison group were screened out for qRT-PCR verification of expression levels. Figure S3: Heatmap of differential metabolite relative content between different groups in negative mode. The first three columns were the three replicates of the experimental group, and the last three columns were that of the control group. Red represents upregulation, while blue represents downregulation. Figure S4: KEGG enrichment pathways of differential metabolites in different comparison groups. The size of the dot represents the number of differential metabolites enriched in different pathways, and their color represents the significance level of the difference. The ratio represents the ratio of the number of differential metabolites annotated to the KEGG pathway to the total number of differential metabolites. The differential metabolites in positive and negative mode enriched pathways were present. Figure S5: The transcription level of structural genes involved in the SA biosynthesis pathway based on MapMan. Green represents upregulation, while red represents downregulation. Figure S6: “Xiangfei” berries under the continuous treatment of UV-C exceeded 10 days. Table S1: General overview of transcriptome data. Table S2: Novel genes identified in the transcriptome. Table S3: List of primer sequences used in qPCR analysis. Table S4: General overview of metabolite data. Table S5: General overview of flavonoids. Table S6: Flavonoids with significant differences between different groups. Table S7: Top three differential metabolites between different groups.
