# Supplementary material for: Transcriptomic and Metabolomic Basis of Short- and Long-Term Post-Harvest UV-C Application in Regulating Grape Berry Quality Development
Source: Foods. 2021 Mar 16;10(3):625. doi: 10.3390/foods10030625 (PMC8001394; doi:10.3390/foods10030625)
Supplement: Supplementary file 1 [file foods-10-00625-s001.zip › Supplementary materials/Supplemental Figure1-6.doc]

**Supplemental Fig. 1** The effect of UV-C on the appearance of grape berries.

**Supplemental Fig.2** Comparison of the gene transcription level detected by RNA-sequencing and qRT-PCR. The 13 differentially expressed genes shared by each comparison group were screened out for qRT-PCR verification of expression levels.

**Supplemental Fig.3** Heatmap of the relative content of differential metabolites between different groups in negtive mode. The first three columns were the three replicates of the experimental group, and the last three columns were that of the control group. Red represents the up-regulation while blue represents the down-regulation.

**Supplemental Fig.4** KEGG enrichment pathways of differential metabolites in different comparison groups. The size of the dot represents the number of differential metabolites enriched in different pathways, and their color represents the significance level of the difference. Ratio represents the ratio of the number of differential metabolites annotated to the KEGG pathway to the total number of differential metabolites. The differential metabolites in positive and negative mode enriched pathways were present, respectively.

**Supplemental Fig.5** The transcription level of structural genes involved in SA biosynthesis pathway based on Mapman. Green represents the up-regulation while red represents the down-regulation.

**Supplemental Fig. 6** ‘Xiangfei’ berries under the continuous treatment of UV-C exceeded 10 days.
