# Supplementary material for: Transcriptomic and Metabolomic Basis of Short- and Long-Term Post-Harvest UV-C Application in Regulating Grape Berry Quality Development
Source: Foods. 2021 Mar 16;10(3):625. doi: 10.3390/foods10030625 (PMC8001394; doi:10.3390/foods10030625)
Supplement: Supplementary file 1 [file foods-10-00625-s001.zip › Supplementary materials/Supplemental Table 3 List of primer sequences used in qPCR analysis.doc]

**Supplementary Table 3** List of primer sequences used in qPCR analysis

| Accession number/Gene name | Primer sequence（5'-3'） | |
| --- | --- | --- |
| VIT_01s0011g05150 | Forward | ATGGCTTCTGCTGATAAGTT |
|  | Reverse | CTCGTCTATGGCTTCTACAA |
| VIT_18s0001g06030 | Forward | CCATCCAACGCTCCATAC |
|  | Reverse | TGTCTGAACTTCGCAATGA |
| VIT_18s0001g06180 | Forward | CCATCCAACGCTCCATAC |
|  | Reverse | TGTCTGAACTTCGCAATGA |
| VIT_17s0000g03600 | Forward | CGGAAGAAGGAGGAGAGT |
|  | Reverse | CATACAGCCACCACAAGT |
| VIT_03s0038g03400 | Forward | ATCATTCAAGACAGCCTTCT |
|  | Reverse | CCACATTCAACACCTCCAT |
| VIT_18s0001g05030 | Forward | TTCGTCAACACCAACAGAT |
|  | Reverse | CGTTCTTCAGAGTCCAGTAA |
| VIT_00s0301g00080 | Forward | GAGGTCAGGTGAAGATGG |
|  | Reverse | AATTGAGTTCTCCGATTGG |
| VIT_18s0001g04280 | Forward | GTGACCAGCAATCCTAAGA |
|  | Reverse | GTTCCTCAGAGACACCATAT |
| novel.183 | Forward | AATTGCCTCTGATGTTGTTG |
|  | Reverse | ACCACTCCTACTGCCATTA |
| VIT_01s0137g00240 | Forward | AATGACGGACTGGTGGAT |
|  | Reverse | AATGGTTGAAGGCAATGGT |
| VIT_13s0074g00700 | Forward | GCTGTAGAATTGGTTGCTAA |
|  | Reverse | CTATGCTTGGCTGGTGAA |
| VIT_16s0039g01670 | Forward | GACTGTGGATCTCAACTTCT |
|  | Reverse | GCAAGGTGAATGGCATCT |
| VIT_10s0071g00230 | Forward | TCAGGCACTATACCAGATTG |
|  | Reverse | TTACATTAGACGACCACAGT |
| *UBIQUITIN1* | Forward | TCTGAGGCTTCGTGGTGGTA |
|  | Reverse | AGGCGTGCATAACATTTGCG |
| *EF1γ* | Forward | CAAGAGAAACCATCCCTAGCTG |
|  | Reverse | TCAATCTGTCTAGGAAAGGAAG |
